# The Use of Virtual Reality in Patients with Eating Disorders: Systematic Review

**DOI:** 10.2196/jmir.7898

**Published:** 2018-04-27

**Authors:** Damien Clus, Mark Erik Larsen, Christophe Lemey, Sofian Berrouiguet

**Affiliations:** ^1^ Department of Mental Health University Hospital of Brest Brest France; ^2^ Black Dog Institute University of New South Wales Sydney Australia; ^3^ Department of Mental Health University Hospital of Brest Université de Bretagne Occidentale Brest France; ^4^ UMR CNRS 6285 Lab-STICC Institut Mines Télécom Atlantique Université Bretagne Loire F-29238 Brest Brest France; ^5^ EA 7479 Soins Primaires, Santé Publique et Registre des cancers de Bretagne Occidentale Department of Mental Health Université de Bretagne Occidentale Brest France

**Keywords:** virtual reality exposure therapy, feeding and eating disorders, binge-eating disorder, anorexia nervosa, bulimia nervosa

## Abstract

**Background:**

Patients with eating disorders are characterized by pathological eating habits and a tendency to overestimate their weight and body shape. Virtual reality shows promise for the evaluation and management of patients with eating disorders. This technology, when accepted by this population, allows immersion in virtual environments, assessment, and therapeutic approaches, by exposing users to high-calorie foods or changes in body shape.

**Objective:**

To better understand the value of virtual reality, we conducted a review of the literature, including clinical studies proposing the use of virtual reality for the evaluation and management of patients with eating disorders.

**Methods:**

We searched PubMed, PsycINFO, ScienceDirect, the Cochrane Library, Scopus, and Web of Science up to April 2017. We created the list of keywords based on two domains: virtual reality and eating disorders. We used the Preferred Reporting Items for Systematic Reviews and Meta-Analyses to identify, select, and critically appraise relevant research while minimizing bias.

**Results:**

The initial database searches identified 311 articles, 149 of which we removed as duplicates. We analyzed the resulting set of 26 unique studies that met the inclusion criteria. Of these, 8 studies were randomized controlled trials, 13 were nonrandomized studies, and 5 were clinical trials with only 1 participant. Most articles focused on clinical populations (19/26, 73%), with the remainder reporting case-control studies (7/26, 27%). Most of the studies used visual immersive equipment (16/26, 62%) with a head-mounted display (15/16, 94%). Two main areas of interest emerged from these studies: virtual work on patients’ body image (7/26, 27%) and exposure to virtual food stimuli (10/26, 38%).

**Conclusions:**

We conducted a broad analysis of studies on the use of virtual reality in patients with eating disorders. This review of the literature showed that virtual reality is an acceptable and promising therapeutic tool for patients with eating disorders.

## Introduction

Patients with eating disorders are characterized by pathological eating habits and a tendency to overestimate their weight and body shape, according to the *Diagnostic and Statistical Manual of Mental Disorders*, Fifth Edition (DSM-5) [1] and the *International Classification of Diseases, Tenth Revision* [2]. Eating disorders are associated with severe medical and psychological outcomes [3], a high risk of death [4,5], and high public health costs [6]. Prevention programs, such as media literacy and psychoeducation, have been put in place to limit these consequences [7]. A large American retrospective survey [8] found prevalences of 0.9% for anorexia nervosa, 1.5% for bulimia nervosa, and 3.5% for binge eating disorder among women and prevalences of 0.3%, 0.5%, and 2%, respectively, among men. These rates were based on the fourth edition of the DSM and would be higher with the application of DSM-5 criteria [9].

International guidelines from the UK National Institute for Health and Care Excellence [10] and the American Psychiatric Association [11] recommend the psychological management of patients with eating disorders, mainly cognitive behavioral therapy (CBT) approaches. The choice of psychotherapy is made according to the patient’s preference; his or her social support, age, and motivation; and the stage of the disease. According to the recommendations of the World Federation of Societies of Biological Psychiatry [12], there is no specific drug therapy for eating disorders. The perceptual component of a person’s body image is addressed during the psychotherapeutic follow-up, with the possible use of photography or video; however, there is a lack of consensus on the media that should be used. Virtual reality (VR) is one possibility.

VR can be defined as a computer technology that reproduces a real or imaginary environment and that simulates the user’s presence in that physical environment, with which the user can interact through engagement of his or her senses (sight, touch, hearing, and smell). VR has been assessed in mental health conditions [13-15] for the rehabilitation of patients with schizophrenia [16], for the treatment of posttraumatic stress disorder symptomatology [17], or for the management of phobic disorders [18]. VR exposes users to interactive 3-dimensional environments that simulate a specific situation [19] and, through guided imagination, overcomes the disadvantages of exposure to a real-life situation, including a possible lack of control over participants’ thoughts and imaginative difficulties [20]. Exposing patients to VR allows delivery of therapy in a form that they may find more acceptable [21]. The virtual environment makes it possible to control the unexpected and to be exposed in a safe environment to certain fears that may be difficult to reproduce in real situations [22], and it affords a greater degree of confidentiality [18]. The main limitations of this technology are the insufficient number of therapists trained in its use, side effects such as “simulator sickness” [23,24], and the high cost of equipment.

Technological advances have made it possible to improve VR techniques, which has led to an increasing number of studies on this subject. However, the latest literature reviews on the use of VR for eating disorders were conducted in 2012 [25-27]. A more recent review of the literature on the use of VR in general psychological treatment of mental illness [13] did not include keywords or derivatives of “eating disorders.”

To better understand the value of this technology, we reviewed the literature including clinical studies proposing the use of VR in patients with eating disorders. The objective of this study was to provide a thorough review of the applications of VR in patients with eating disorders.

## Methods

We used the Preferred Reporting Items for Systematic Reviews and Meta-Analyses (PRISMA) [28] to identify, select, and critically appraise relevant research while minimizing bias.

### Search Strategy

We extensively searched PubMed, PsycINFO, Science Direct, the Cochrane Library, Scopus, and Web of Science up to April 2017. We based the list of keywords on two domains: virtual reality and eating disorders. A search strategy was constructed using the Boolean operators AND and OR and applied in the Medical Subject Heading, title, and abstract fields. The keywords and search strategy we used were (virtual reality OR virtual environment OR immersive reality) AND (eating disorders OR anorexia OR bulimia OR disordered eating OR binge eating disorder) in any language, but referenced in the selected databases and meeting the inclusion criteria. To limit the risk of selection bias, we applied no restrictions in terms of article type or clinical population. We did not include serious games, defined as video games with a pedagogical, diagnostic, or therapeutic interest, in the search terms because these do not necessarily rely on immersive VR technology. We excluded all articles not written in English.

### Study Selection

We included randomized controlled trials and nonrandomized studies. The primary end point was the collection of studies using VR for eating disorders, specifically articles that addressed the use of VR techniques in an eating disorder sample only, in comparison with either another treatment condition or a control group. We excluded studies without a clinical population and theoretical articles presenting an application of VR without final results. We also excluded studies exploring the use of serious games, without the use of VR, for these disorders.

### Data Extraction

We screened each study and extracted data independently using standard forms. The following information was extracted from each study: first author’s last name, publication year, and country; study design; population analyzed; number of patients; evaluation scales used; study objectives; study protocol; and study results.

## Results

**Figure 1:**
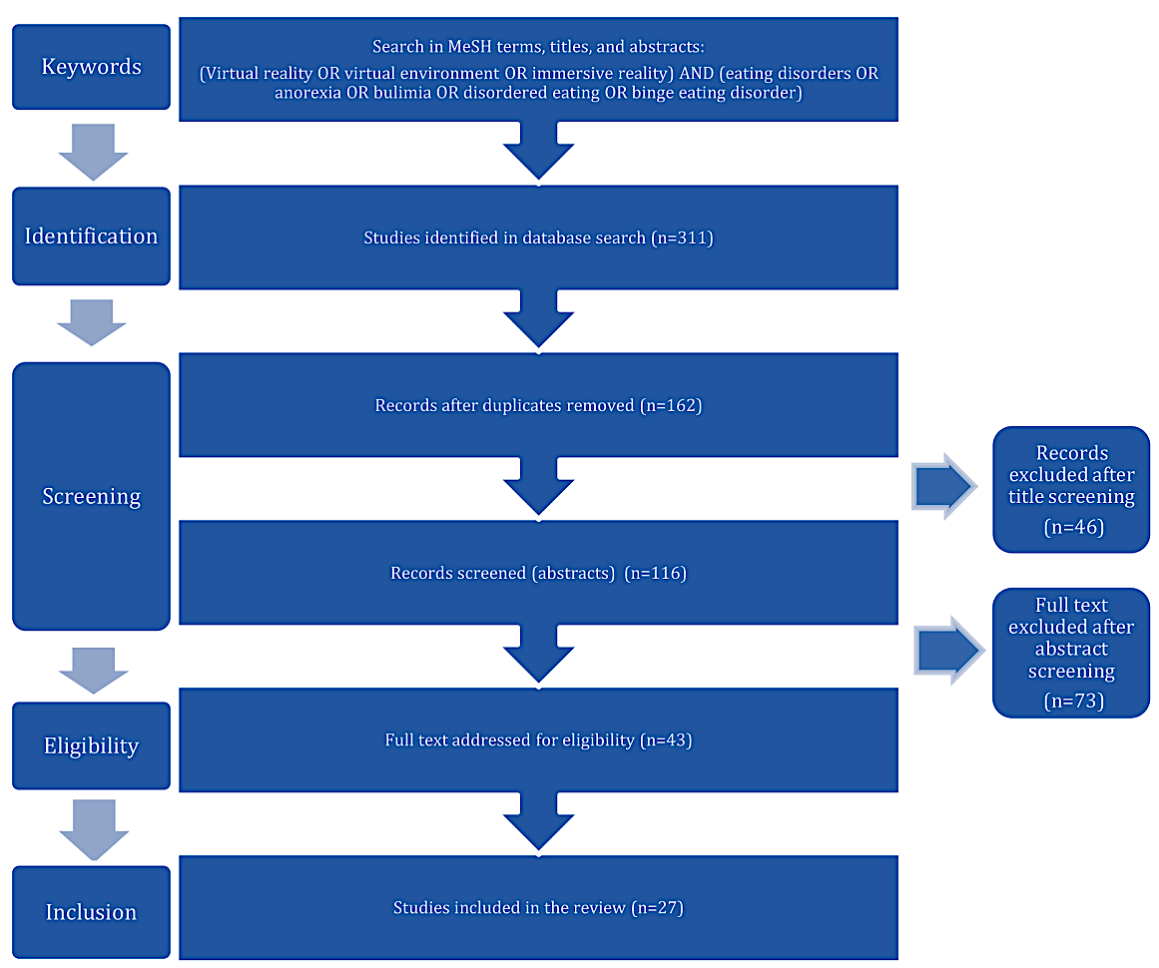
Flow diagram of study selection. MeSH: medical subject headings.

Figure 1 shows the PRISMA flowchart summarizing the stages of the review. The initial database searches identified 311 articles, 149 of which we removed as duplicates. Following review of the titles and abstracts, we excluded 119 articles. We downloaded the remaining 43 articles for full-text review, following which we excluded an additional 17 articles: 7 articles presented a protocol without results; 3 studies evaluated healthy participants; 2 articles were not entirely in English; 2 articles reported studies already included; 2 studies reported preliminary data; and 1 study described a serious game without VR. We analyzed the resulting set of 26 unique studies that met the inclusion criteria.

Multimedia Appendix 1 [29-54] presents the results of this review in a descriptive way and in the form of a table.

### Study Authors, Year of Publication, and Country of Origin

**Figure 2:**
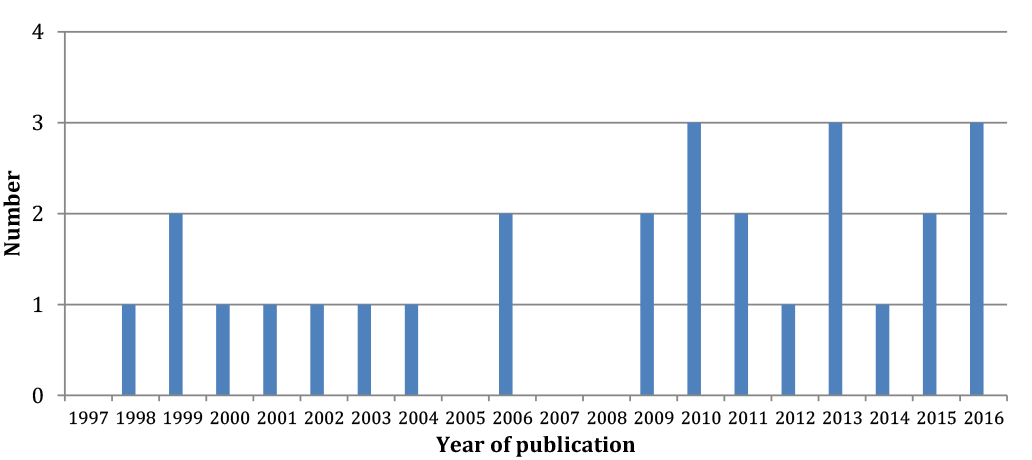
Number of articles included in the review by year published.

The team of Riva et al [29-36] produced approximately one-third of the included articles (8/26, 31%). The last literature reviews on this subject [25-27] included articles that were published up to 2010. Since then, 12 (46%) articles included in our selection were published, suggesting an increase in interest in using these new technologies for patients with eating disorders (Figure 2). All the selected articles were from European teams and 2 countries are the most represented in this literature review: Italy (14/26, 54%) and Spain (11/26, 42%); the remaining study was conducted in the United Kingdom.

### Study Design, Population, and Sample Size

Concerning the study methodologies, 8 studies were randomized controlled trials, 13 were nonrandomized studies, and 5 were clinical trials with only 1 participant. Most articles focused on clinical populations (19/26, 73%), with the remainder reporting case-control studies (7/26, 27%). Most participants were female (24/26, 92%); only 2 studies included mixed samples (male and female). The clinical populations observed in this literature review were heterogeneous from a diagnostic point of view in 15 studies (58%). The other studies targeted specific disordered eating: obese patients without psychiatric comorbidities (n=6, 23%), anorexia nervosa (n=3, 12%), and binge eating disorder (n=3, 12%). The numbers of patients varied from single case studies (5/26, 19%) to larger samples of more than 100 patients (8/26, 31%).

### Technology Used

Most of the studies used visual immersive equipment (16/26, 62%) with a head-mounted display (15/16, 94%). One study used a cubic immersive room with the projection of a virtual environment on the walls (cave system). A total of 7 studies used audio stimuli in combination with visual kinematics to allow user–virtual world interaction and gradual exposure to high-calorie food stimuli associated with chewing sounds or comments on ingested foods. Also, only 1 study used visual-tactile stimulation complemented with immersive material, which included an obese patient to test the effect on her assessment of her body measurements.

### Objectives of the Studies

Two main areas of interest emerged from these studies: virtual work on patients’ body image (7/26, 27%) and exposure to virtual food stimuli (10/26, 38%). Some study protocols analyzed both of these fields (6/26, 23%). A total of 15 (58%) studies had a primary therapeutic objective; 11 (42%) studies evaluated the users’ tolerance of the protocol and their emotional reactions during the VR immersion. Multimedia Appendix 1 presents the main results.

## Discussion

### Principal Findings

We conducted a broad analysis of studies on the use of VR in patients with eating disorders. This review of the literature showed an increase in interest in using these new technologies for patients with eating disorders, with 26 articles. Since the last reviews of the literature dealing with this topic, 12 articles have been published, testifying to the always-present interest in this technology. Commercialized VR technology is increasingly accessible to the general public, and medical research, especially in mental health, is increasingly using this new tool, with diagnostic, therapeutic, and preventive aims. The use of VR in the evaluation or treatment of patients with eating disorders is being led by European teams.

It is difficult to interpret the results of this research because of the heterogeneity of the populations studied, the studies’ objectives, and the content of the VR protocols. Many studies drew conclusions without differentiating the subtypes of disordered eating, with low sample sizes.

This review of the literature nevertheless allows us to discuss certain aspects of this field of investigation.

### Virtual Reality, an Acceptable and Effective Therapeutic Tool

Some studies with medium- and long-term follow-up showed less loss to follow-up with a VR protocol compared with other groups [38,54]. This can be explained by the attractiveness of new technologies and exposure to stimuli in a virtual environment in the presence of a therapist. Starting therapy could help foster a therapeutic alliance, an active participation in a process of change. Several studies observed patients’ increased motivation for change [31,32,37,55].

The use of a VR module in addition to CBT showed greater efficacy in the main variables analyzed in comparison with control groups [36,52] or CBT alone [29,30,35,38,43,44,54]. European [56] and American [11] guidelines recommend the use of CBT in the psychological care of bulimia nervosa and binge eating disorder. CBT is considered one of the solutions for psychological interventions in patients with anorexia nervosa [11,56,57]. CBT approaches are recommended for patients with obesity [58]. Thus, CBTs are valid therapies in for patients with eating disorders and are viable for a comparative judgment of the effectiveness of a new approach.

These results are, however, to be qualified by certain limits cited by this research. Samples of the studies were small due to recruitment difficulties presented by the low frequency of these disorders. The difficulties that certain populations, such as those with anorexia nervosa, have in engaging with care may also explain this result. Most of this research evaluated a female population, which is explained by the female predominance of eating disorders [3]. Also, the lack of controlled randomized clinical trials leads us to be cautious in interpreting these results. All of the studies were developed by European teams, notably by Riva’s team. It is surprising that there were no studies of North American origin due to the socioeconomic impact of these pathologies on this continent [6]. Similarly, most of the technologies used to enable immersion in the virtual environment have been developed in the United States. The predominance of Riva’s research team may also be related to an interpretation bias in the final analysis of the articles.

### Theory of Maintenance of the Perturbations of Body Image

Some studies [37,42] advanced assumptions on the maintenance of pathological disorders. The hypothesis developed to explain the perturbations of body image in patients with eating disorders is the allocentric lock hypothesis [59,60]. Body image disorders in patients with eating disorders are related to a deficiency in their ability to update their negative body image stored in their memory (allocentric function) with sensory motor and proprioceptive inputs in real time (egocentric function). In patients with anorexia nervosa, this deficit is related to an involvement of the lower parietal lobe and precuneus, the gyrus of the inner surface of the parietal lobe of the cerebral cortex [61]. This hypothesis could also apply in patients with obesity [62]. The use of VR could make it possible to unblock this transmission [63]. The theory of objectification as a specific cognitive process is cited to understand these perturbations: a person internalizes an objectified self-image when they use a reference allocentric frame (observer mode) to recall the events in which they evaluate themselves on the basis of body appearance [64].

### Virtual Environments for Patients’ Disorders

The validity of using a virtual environment in a population can be judged by the individual’s emotional reactions. Unique exposure to virtual food stimuli increases anxiety and changes mood [45,47,49-51,53], reproducing physiological reactions to a real situation. Similar emotional reactions were found between exposures to real and virtual foods [49]. The repetition of VR sessions with modules of exposure to food stimuli and silhouettes of the patient or mannequin reduces negative emotions [29,34,46,54], by progressive attenuation of the anxiogenic response.

Nevertheless, one may question the relevance of the results of certain studies [50,51,53] whose objective was to evaluate emotional reactions in a virtual environment. The use of an evaluation tool such as the Barcelona Depression Questionnaire, a self-questionnaire of low scientific validity measuring variations in depressed mood, warrants interpreting these conclusions cautiously.

### Limitations

This analysis of the literature had some limitations. It excluded studies with a nonclinical population—these typically described the use of VR with objectives of acceptability and tolerance, to be reproduced later with clinical samples. We excluded from the analysis those articles proposing a VR study model on eating disorders without final results, as well as meeting abstracts and a protocol using a serious game, without immersive material, to evaluate impulsivity in bulimic patients [65].

In the bibliographic search, we did not use the term obesity. Indeed, according to international classifications recognized in psychiatry [1], obesity without psychiatric comorbidities is not part of the spectrum of eating disorders. Certain psychiatric pathologies belonging to this spectrum (binge eating disorder) can result in weight gain up to obesity [3]. The lifetime prevalence of obesity in patients with eating disorders is estimated at 28.8% [66]. In the final analysis, because of this frequent association, we included articles with obese participants.

### Conclusion

VR is increasingly being applied in the evaluation and management of patients with eating disorders, with a recent increase in articles being published. This technology, when accepted by this population, allows patients to be immersed in virtual environments that are adapted to their psychological state, causing reactionary emotions as in real life, in a safe environment, under the supervision of a therapist. Upcoming technical improvements of VR will also provide a better sense of presence. Overall, VR techniques enable the evaluation of pathological eating behaviors and body image distortions. In addition to CBT, use of VR techniques by patients with eating disorders decreased their negative emotional responses to virtual food stimuli or exposure to their body shape.

## Appendix

Multimedia Appendix 1 Details of the reviewed articles.

## Abbreviations

CBT: cognitive behavioral therapy
DSM: Diagnostic and Statistical Manual of Mental Disorders
PRISMA: Preferred Reporting Items for Systematic Reviews and Meta-Analyses
VR: virtual reality
