# Supplementary material for: The Use of Virtual Reality in Patients with Eating Disorders: Systematic Review
Source: J Med Internet Res. 2018 Apr 27;20(4):e157. doi: 10.2196/jmir.7898 (PMC5948410; doi:10.2196/jmir.7898)
Supplement: Multimedia Appendix 1 [file jmir_v20i4e157_app1.pdf]

Articles included in the review of the literature

Authors, year, country	Design	Population	n	Evaluation Scales	Objectives	Protocol	Results
**Serino S, 2016, Italy [37]**	Case	1 female obese patient	1	- Evaluation task of the width, circumference of the abdomen, shoulders and hips<br>- Tactile distance estimation task on the abdomen	Presentation of a VR technique with tactile stimulation and case study	Number of VR sessions: NS<br><br>Immersive material (HDM) with synchronous tactile stimulation (horizontal touch) / asynchronous (fixed touch) on the abdomen, first person view on the abdomen<br><br>Pre / post treatment evaluation	Pre-treatment evaluation:<br>- Underestimation of the width of body parts<br>- Circumferential overestimation<br><br>Post-treatment evaluation:<br>- Reduction of the underestimation of the width of body parts<br>- Reduction of circumferential overestimation<br>- Tactile estimation task: heterogeneity of results<br>- Motivation for change<br>- Weightloss
**Manzoni GM, 2016, Italy [38]**	Case<br><br>RCT	163 female obese patients	163	- Weight<br>- DIET<br>- BSS<br>- BIAQ<br>- CDRS	Assessment of the short- and long-term effectiveness of the ECT module.	3 conditions:<br>- Control group: standard treatment (diet + physical activity)<br>- CBT group: standard treatment and CBT (5 weekly group sessions and 10 biweekly individual sessions)<br>- VR group: standard treatment and VR + CBT (5 weekly group CBT sessions and 10 individual bi-weekly VR + CBT sessions (ECT))<br><br>Immersive Material (HDM), NeuroVR<br><br>Pre / Post Treatment and 1 Year Assessment	Post-treatment evaluation:<br>- No significant differences between the 3 groups; significant weight loss in all groups<br>- Improvement of dietary behavior (DIET), body satisfaction (BSS, BIAQ, CDRS) with no significant difference between the 3 groups. Best results in the VR group.<br><br>Evaluation at 1 year:<br>- Drop-out in the control group (n = 21) > CBT group (n = 14)> VR group (n = 10). Significant association between drop-out and experimental conditions<br>- Significant weight loss only in the VR group<br>- Significant weight gain only in the control group<br>- Likelihood of maintaining or losing weight at 1 year in VR condition compared to control or CBT conditions.
**Perpina C, 2016, Spain [39]**	Case-control	20 female patients ED:<br>- 8 AN restrictive<br>- 2 AN purge<br>- 4 BN<br>- 3 EDNOS purge<br>- 3 EDNOS restrictive<br><br>- 19 female obese patients<br><br>- 23 controls	62	- Self-questionnaire exploring the choice of "forbidden" virtual food<br>- RJPQ<br>- ITC-SOPI	Comparison between ED and obese groups on the judgment of the reality of a virtual environment designed to normalize their mode of feeding Study of the variables predicting the reality of the ingestion of a virtual "forbidden" food.	One VR session<br><br>Non-immersive material, sound effects<br><br>Post VR session evaluation	- The highest scores of ED group in emotional involvement, attention, judgment of reality, presence and negative feelings of the "forbidden" food.<br>- Weakest scores of obese group in the judgment of reality and presence, satisfaction, sense of physical space in the virtual environment. Intermediate position for obese group in virtual reality food<br>- Reality of ingestion of the "forbidden" virtual food predicted by the level of engagement in the virtual environment (ITC-SOPI) for ED patients, and the level of attention (RJPQ) for obese patients.
**Pla-Sanjuanelo J, 2015, Spain [40]**	Case-control	40 patients ED:<br>- 17 BED<br>- 23 BN<br>- sex mixed	118	- FCQ-T/S<br>- Analogue scale (0 - 100) assessing craving level	Evaluate whether exposure to virtual food signals produces compatible levels of	One VR session<br><br>Non immersive material, 3D glasses	- High levels of Trait craving and State craving predict a greater desire to eat in the virtual environment in controls and patients.<br>- Only significant correlation between the craving state (FCQ-S) and the virtual craving experience in patients.

**Ferrer-Garcia M, 2015, Spain [41]**		78 controls		- DEBQ : self-questionnaire exploring three different types of eating habits :<br>- DEBQ-EX: consumer “external”<br>- DEBQ-EM: consumer “emotional”<br>- DEBQ-RE : consumer " “restrictive”<br>- Analogue scale (0 - 100) assessing craving level	craving state and craving traits<br><br>Evaluation of the relationship between eating habits and the desire for food after exposure to virtual food stimuli<br>Evaluation of the utility of VR technology as an exposure technique to induce food cravings in "external" consumers.	Post VR exposure assessment	- "External" consumer mode more sensitive to virtual food stimulation. This eating mode predicts the occurrence of craving.<br>- Increased levels of "external", "emotional", "restrictive" consumer in ED patients
**Serino S, 2015, Italy [42]**	Case-control	22 female patients ED:<br>- 11 AN<br>- 11 BN<br><br>22 female controls	44	- Corsi Block Test-Span and Supraspan<br>- Money Road Map<br>- Manikin's Test<br>- Judgment of Line Orientation	Study of potential deficiencies in spatial capabilities and spatial processing.<br>Allocentric / egocentric memory.	One VR session<br><br>Immersive equipment (CAVE)<br><br>Pre / post protocol evaluation	- Results significantly lower in ED group compared to control group in the short and long term spatial memory (Corsi Block Test-Span, Supraspan), navigation (Money Road Map), mental rotation (Manikin's Test) and visuo-spatial capabilities (Judgment of Line Orientation).<br>- Defective allocentric representation for ED patients.
**Cesa GL, 2013, Italy [43]**	Case	90 female obese patients with BED	90	- BSS<br>- BIAQ<br>- CDRS<br>- Weight	Comparison of short- and long-term efficacy of CBT + VR (ECT), CBT alone, usual hospital care on weight loss, maintenance of weight loss, remission, improvement in body satisfaction.	<u>3 conditions:</u><br>- <u>VR group:</u> 15 sessions CBT + VR (ECT) for 5 weeks + routine care<br>- <u>CBT group:</u> 15 CBT sessions for 5 weeks + routine care<br>- <u>Control group:</u> routine care<br><br>Immersive Material (HDM), NeuroVR<br><br>Evaluation 1 week after the start of treatment, after treatment completed and at one year.	- CBT + VR technique only effective on weight loss at 1 year. Control group: weight recovery greater than loss during hospitalization at 1 year evaluation.<br>- Recurrent binge eating in the three groups at 1 year of follow-up but maintaining a low monthly rate in VR and CBT groups.

**Marco JH, 2013, Spain [44]**	Case<br><br>RCT	34 female patients ED:<br>- 17 BN<br>- 12 EDNOS<br>- 5 AN	34	- BAT<br>- BIATQ<br>- BASS<br>- BITE<br>- SIBID<br>- EAT-40<br>- Weakly evaluation symptoms	Evaluation of the short- and long-term effectiveness of a CBT and VR module.	2 conditions:<br>- CBT group:<br>- BN: 19 group sessions<br>- AN: 23 individual sessions<br><br>- VR group:<br>15 CBT sessions in group + 8 sessions of individual psychotherapy with VR technique<br><br>Immersive Equipment (HDM)<br><br>Pre / post treatment evaluation and follow-up at 1 year.	- Significant improvement body image in both groups in post-treatment and 1-year<br>- Significant differences in effectiveness in favor of the VR group, on body attitudes (BAT), negative thoughts related to the body (BIATQ), body satisfaction (BASS), discomfort in situations related to body image (SIBID), bulimic symptomatology (BITE).
**Perpina C, 2013, Spain [45]**	Case-control	22 female patients ED :<br>- 11 AN<br>- 4 BN<br>- 7 EDNOS<br><br>37 female controls	59	- BDI-II<br>- BAI<br>- RS<br>- Questionnaire created to evaluate the reality of the virtual environment<br>- RJPQ<br>- ITC-SOPI	Study of the clinical validation of a virtual environment intended to normalize eating habits of ED patients	One VR session<br><br>Non-immersive material, sound effects<br><br>Pre/ post VR session evaluation	- Emotional response higher in ED group (anxiety (BAI), depressed mood (BDI) and food-related thoughts (RS))<br>- Sense of presence (RJPQ, ITC-SOPI) felt by the 2 groups, without differences.<br>- Increased levels of eating, fear, avoidance, feeling of weight gain during the experiment and greater attention, emotional involvement, side effects for ED patients after virtual feeding.
**Riva G, 2012, Italy [34]**	Case	1 female obese patient, after bariatric surgery	1	- BDI<br>- STAI<br>- BSQ<br>- BULIT<br>- TFEQ	Study of an VR protocol (ETC) on an obese patient after bariatric surgery	5 CBT weekly group sessions and 10 individual bi-weekly VR sessions (ECT); 15 sessions in 6 weeks<br><br>Immersive Material (HDM), NeuroVR<br><br>Pre / post protocol evaluation	- Improved depressive mood (BDI), anxiety (STAI), body image (BSQ)<br>- Improved food control (BULIT, TFEQ)<br>- Improvement of social relations
**Cardi V, 2012, England [46]**	Case	1 female, restrictive AN patient	1	- EDE-Q<br>- DASS<br>- Weight<br>- Questionnaire created evaluating the negative thoughts, emotions, acceptability, utility perceived by the patient.	Study of an VR module of progressive exposure to virtual food	7 weekly VR sessions<br><br>Non-immersive material, sound effects<br><br>Pre / post treatment evaluation	- Lower levels of anxiety, avoidance and fear related to food (DASS).<br>- Food diversification in life<br>- Decreased ED symptomatology (EDE-Q), improved mood, self-esteem (DASS).<br>- Improvement of family and social relations<br>- Weight recovery
**Ferrer-Garcia M, 2011, Spain [47]**	Case	71 female patients ED:<br>- 49 AN<br>- 22 BN	71	- Analogue scale to measure the level of discomfort<br>- Presence Questionnaire	Study of variables influencing the emotional response (discomfort) of ED patients in VR	One VR session, randomization of virtual environments<br><br>Non immersive equipment, headphones	- Highly caloric food and social situation lead to the highest levels of discomfort<br>- Emotional reaction proportional to the severity of eating disorders and the sense of presence in the virtual environment.

				- EAT-26	environments	Pre / post VR session evaluation	
**Gutierrez-Maldonado J, 2010, Spain [48]**	Case-control	85 female patients ED:<br>- 49 AN<br>- 22 BN<br>- 14 EDNOS<br><br>108 female controls	193	- BIAS	Study the effect of VR exposure on emotionally significant situations for ED patients on the stability of body image distortion and dissatisfaction	2 VR sessions:<br>- 1st: immersion in a neutral environment<br>- 2nd: immersion in the 4 remaining environments, randomization of the virtual environments.<br><br>Non immersive equipment, headphones<br><br>Pre / post protocol evaluation	- Increased levels of body image distortion (BIAS) and body dissatisfaction (BIAS) in ED patients after ingesting high calorie foods.<br>- Correlation between body distortion (overestimation) and the presence of persons in BN patients; No correlation for AN and EDNOS patients<br>- Correlation between body dissatisfaction and food variable in AN and EDNOS patients; No correlation for BN patients<br>- No correlation between body dissatisfaction and the presence of persons variable for AN, EDNOS and BN patients.
**Gorini A, 2010, Italy [49]**	Case-control	20 female patients ED :<br>- 10 AN<br>- 10 BN<br><br>10 female controls	30	- STAI-S<br>- Visual Analog Scale Assessing Anxiety<br>- ITC-SOPI<br>- Cardiac frequency<br>- Respiratory rate<br>- Skin conductance	Study of emotional reactions after exposure to real, virtual food and photographs.	One VR session, randomization order and exposure conditions.<br><br>Immersive Material (HDM), NeuroVR<br><br>Pre / post exposure assessment	- Comparative emotional response in patients with exposure to real and virtual food, higher than that obtained by photographs.<br>- No significant differences in emotional reactions between AN and BN.<br>- Significant effect between the degree of presence of subjects in VR condition and their level of anxiety: the greater the impression of presence, the higher the level of anxiety.
**Ferrer-Garcia M, 2010, Spain [50]**<br><br><br><br><br>**Ferrer-Garcia M, 2009, Spain [51]**	Case-control	85 female patients ED<br>- 49 AN<br>- 22 BN<br>- 14EDNOS<br><br>108 female controls	193	- STAI-S<br>- CDB<br>- BIAS	Study whether anxiety and depressed mood produced by virtual reality exposure influence body image distortion and dissatisfaction in ED patients and controls.<br><br><br>Evaluate the validity of virtual environments representing emotionally intense situations for ED patients to cause different changes in mood and anxiety levels in patients and controls.	One VR session, randomization of virtual environments<br><br>Non immersive equipment, headphones<br><br>Pre / post VR session evaluation	- Increased correlation between anxiety (STAI) or depressive mood (CDB) and body distortion (BIAS) in ED patients versus controls<br>- Increased correlation between anxiety or depressed mood and body dissatisfaction (BIAS) in ED patients compared to controls<br>- The strongest significant relationship between depressed mood and body dissatisfaction, especially in virtual environments with high-calorie foods<br>- Association of anxiety and predictive depressive mood of body distortion and body dissatisfaction in ED patients<br><br><br>- Higher levels of anxiety (STAI) and depressed mood (CDB) in the 5 environments compared to the neutral environment for ED patients<br>- Higher levels of anxiety and depressed mood after ingesting high-calorie foods versus low-calorie foods for ED patients<br>- Variable presence of other people modulating the effect of food only in group BN<br>- Higher levels of anxiety and depressed mood when exposing the body (swimming pool) to the neutral environment in ED group
**Manzoni GM, 2009, Italy [52]**	Case<br><br>RCT	60 female obese patients	60	- BDI<br>- WELSQ<br>- STAI<br>- EOQ	Evaluation / comparison of the effectiveness of a relaxation program	<u>3 conditions:</u><br>- <u>VR group:</u> 12 sessions of relaxation VR + music during 3 weeks<br>- <u>Imagination group:</u> 12 relaxation	<u>Post-treatment evaluation:</u><br>- Significant improvement in food self-control (WELSQ), depressed mood (BDI) in the RV and Imagination group; No significant difference between the 2 groups; No significant change in the control

				- Weight	with VR sessions	sessions in vivo for 3 weeks<br>- Control group<br><br>Immersive Material (HDM), NeuroVR<br><br>Pre / post treatment evaluation and follow-up at 3 months	group.<br><br>Evaluation at 3 months:<br>- Significant improvement in the effectiveness of food self-control (WELSQ), depressed mood (BDI), anxiety (STAI) in VR and Imagination groups compared to control group.<br>- Decreased emotional intake (EOQ) in VR and Imagination groups compared to control group; Significant difference in favor of VR group / Imagination group<br>- No significant difference for weight loss between the 3 groups.
**Gutierrez-Maldonado J, 2006, Spain [53]**	Case<br><br>RCT	30 female patients ED:<br><br>- 17 AN<br>- 11 BN<br>- 2 EDNOS	30	- STAI<br>- CDB<br>- Presence Questionnaire	Evaluation of anxiety and depressed mood after exposure to different types of virtual foods and the presence of other virtual people	One VR session, randomization of virtual environments<br><br>Hardware NS<br><br>Pre/ post VR session evaluation	- Highest level of anxiety (STAI) in the kitchen with high-calorie foods, in the restaurant with high-calorie foods and at the swimming pool in comparison with the neutral environment.<br>- More depressive mood (CDB) in virtual places with high calorie foods.<br>- Significant differences in anxiety between rich / low calorie foods<br>- No significant difference according to the presence or not of persons except for the anxiety at the swimming pool.<br>- No variable interactions food + people on anxiety and depressed mood.<br>- No significant difference was found for the variable "feeling of presence" in the virtual environment on anxiety and depressed mood in different environments.
**Riva G, 2006, Italy [35]**	Case<br><br>RCT	211 female obese patients	211	- Weight<br>- DIET<br>- STAI<br>- WELSQ<br>- BSS<br>- BIAQ<br>- CDRS	Comparative study of the effectiveness of the ECT module	4 conditions:<br>- Control group: no treatment<br>- Dietetic group: 5 weekly sessions in nutritional counseling, diet, physical activity<br>- CBT group: 5 group psychological sessions + 10 individual psychological sessions + dietary group content<br>- VR group: 5 psychological sessions in group + 10 individual sessions VR with psychologist (ECT) + content group diet<br><br>Immersive equipment (HDM), VREDIM<br><br>Pre / post treatment evaluation and follow-up at 6 months	Pre / post treatment evaluation:<br>- Significant weight loss in all 3 groups with treatment.<br>- Significant reduction in anxiety (STAI), improvement of dietary behaviors (DIET, WELSQ), body satisfaction (BSS, BIAQ, CDRS) in all 3 groups with treatment.<br>- No significant difference between the 3 groups with treatment; Best results in VR group.<br><br>Evaluation at 6 months:<br>- Significant weight loss in the 3 groups with treatment, with no significant difference between the groups.<br>- Results on the weight loss greater than 10% relative to the initial weight, on the maintenance or the loss of weight after the end of the treatment, on a weight equal to or greater than the initial weight: significant difference between the 3 groups. Results for VR group<br>- Significant improvement in the results of body satisfaction (BSS, BIAQ), feeding behavior (WELSQ) in VR group compared to CBT and dietary groups
**Riva G, 2004, Italy [29]**	Case<br><br>RCT	120 female patients :	120	- STAI<br>- BDI-II<br>- RSEQ	Description of a new VR tool associated with CBT sessions	4 conditions:<br>- Control group: no hospital management	- BN and EDNOS data excluded from statistical analyzes due to too small samples

		- 68 obese<br>- 36 BED<br>- 12 BN<br>- 3 EDNOS<br>- 1 NS		- RAS<br>- DIET<br>- WELSQ<br>- URICA<br>- BSS<br>- BIAQ<br>- CDRS	(ECT) and comparative study	- Dietetic group: weekly psychological support + 1 individual diet session + 4/6 group diet sessions + physical activity<br>- CBT group: 5 group psychological sessions + 10 individual psychological sessions + 1 individual diet session + 4/6 group diet sessions + physical activity<br>- VR group: 5 group psychological sessions + 10 individual VR sessions with psychologist (ECT) + 1 individual diet session + 4/6 group diet sessions + physical activity<br><br>Immersive material (HDM), VREDIM: 10 sessions<br><br>Pre / post treatment evaluation	BED patients:<br>- Decreased anxiety (STAI) in VR and dietetic group<br>- Reduction depressive mood (BDI) in VR and CBT groups; Amendment of depressive symptomatology only in VR group<br>- Improved self-esteem (RSEQ) in all groups except the control group<br>- Improvement of the patient's social skills (RAS) in VR group<br>- Improved food self-control (DIET, WELSQ) in all groups except the control group; Best results for VR group<br>- Improved body image (BSS, BIAQ, CDRS) more effective in VR group<br>- Reduction of weight in all groups except in the control group<br><br>Obese patients:<br>- Decreased anxiety (STAI) in VR, CBT and dietetic groups<br>- Decreased depressive mood (BDI) in VR and CBT groups; Amendment of depressive symptomatology only in VR group<br>- Improved self-esteem (RSEQ) in VR and CBT groups<br>- Improvement of social skills (RAS) of the patient in VR group<br>- Improved food self-control (DIET, WELSQ) in all groups except the control group; Best results for VR group<br>- Improved body image (BSS, BIAQ, CDRS) more effective in VR group<br>- Reduction of weight in all groups except the control group
**Riva G, 2003, Italy [30]**	Case<br><br>RCT	36 female patients BED	36	- STAI<br>- BDI-II<br>- RSEQ<br>- RAS<br>- DIET<br>- WELSQ<br>- URICA<br>- BSS<br>- BIAQ<br>- CDRS	Description of a new VR tool associated with CBT sessions (ECT) and comparative study	4 conditions:<br>- Control group<br>- Dietetic group: 5 weekly diet sessions as a group + physical activity<br>- CBT group: 5 group psychological weekly sessions + 10 biweekly individual psychological sessions + 5 weekly diet sessions in group + physical activity<br>- VR group: 5 weekly psychological sessions in group + 10 individual VR sessions biweekly with psychologist (ECT) + 5 weekly diet sessions in group + physical activity<br><br>Immersive material (HDM), VREDIM: 10 sessions<br><br>Pre / post treatment evaluation and at 6 months	Results before / after Treatment:<br>- Decreased anxiety (STAI) in VR and dietetic groups<br>- Decreased depressive mood (BDI) in VR and CBT groups; Amendment of depressive symptomatology only in VR group<br>- Improved self-esteem (RSEQ) in all groups except the control group<br>- Improvement of the patient's social skills (RAS) in VR group<br>- Improvement of food control (DIET, WELSQ) in all groups except the control group; Best results for VR group<br>- Improved body image (BSS, BIAQ, CDRS) more effective in VR group<br>- Reduction of weight in all groups except the control group<br><br>Results at 6 months:<br>- Better efficacy of VR group on bulimic behavior (EDI 2) and on body image (BSS, BIAQ, CDRS)<br>- 77% of VR group without binge eating / 56% for CBT group / 22% dietetic group<br>- No significant differences between the 3 intervention groups on weight loss (weight gain 0.5 - 1.5kgs)
**Riva G, 2001, Italy [36]**	Case	28 female obese	28	- DIET	Description of a new	2 conditions:	VR group:

	RCT	patients		- STAI<br>- AI<br>- WELSQ<br>- URICA<br>- BSS<br>- BIAQ<br>- FRS<br>- CDRS	VR tool associated with CBT sessions (ECT) and comparative study	- VR group: 7 VR sessions associated CBT + diet + physical activity<br><br>- Control group: diet + physical activity + group psycho-dietetic sessions (3 times a week)<br><br>Immersive equipment (HDM), VREDIM<br><br>Pre / post treatment evaluation	- Reduced body dissatisfaction (BSS), decreased level of anxiety (STAI, AI anxiety)<br>- Increased self-control, decreased excessive dietary intakes (WELSQ, DIET)<br>- Weightloss<br><br>Comparison RV and control group:<br>- Significant differences in favor of VR group (BSS Total score, DIET Positive Social score, AI Ability, and Anxiety score). Trends for WELSQ Total score and for the URICA Maintenance score.<br>- VR group: Reduced physical dissatisfaction, level of anxiety.
**Riva G, 2000, Italy [33]**	Case	- 25female patients BED<br><br>- 18 female obese patients<br><br>- 14 female patients EDNOS	57	- BSS<br>- BIAQ<br>- FRS<br>- CDRS	Description of a new VR tool associated with CBT sessions	5 individual VR sessions associated with CBT, biweekly sessions + weekly psychological support + psycho-dietetic sessions biweekly in group + diet<br><br>Immersive Material (HDM), VEBIM-2<br><br>Pre / post treatment evaluation	BED group:<br>- Reduction of body dissatisfaction (BSS, FRS, CDRS)<br>- Improvement of social activities (BIAQ Social Activities)<br>- Reduction in the use of clothing concealing their forms (BIAQ Clothing)<br><br>Obese group:<br>- Reduction of body dissatisfaction, better representation of their members<br>- Improvement of social activities<br>- Reduction in the use of clothing concealing their forms<br><br>EDNOS group:<br>- Reduction of body dissatisfaction, less reduction in comparison with obese group and BED<br>- Improvement of social activities<br>- Reduction in the use of clothing concealing their forms
**Perpina C, 1999, Spain [54]**	Case	13 ED patient :<br><br>- VR group: 4 AN et 4 BN<br><br>- Not VR group : 3 AN et 2 BN	18	- BDI<br>- PANAS<br>- EAT<br>- RS<br>- BITE<br>- EDI-2<br>- BSQ<br>- BIAQ<br>- BAT<br>- BES<br>- BIATQ<br>- ASI<br>- SIBID<br>- BASS<br>- Body Interference<br>- Fear of putting on weight	Study of the efficacy of an VR treatment on body image disorders in ED patients Comparison with conventional treatment	2 conditions:<br>- VR group: 6 weekly VR sessions + 6 weekly relaxation sessions + 8 CBT group sessions<br><br>- Not VR group: 6 sessions of relaxation weekly + 8 sessions CBT in group<br><br>Immersive Equipment (HDM)<br><br>Pre / post treatment evaluation	Comparison VR / not VR group:<br><br>- Significant improvement in depressive mood (BDI) and anxiety (PANAS), body image satisfaction (BAAS, BSQ), body acceptance (SIBID, BIAQ, BIATQ), less fear to gain weight, to weigh themselves.<br>- Reduction of the difference between the actual weight and the estimated weight; Desired weight closer to target medical weight<br>- No significant differences found in the general ED scales (EAT, RS, BITE, EDI-2)<br>- 5 stops protocol in not VR group
**Riva G, 1999, Italy [32]**	Case	1 female patient AN	1	- BSS	Description of a new	- 5 individual VR sessions associated	- Increased awareness of the body

**Riva G, 1998, Italy [31]**		binge-eating/ purging type		- BIAQ<br>- CDRS<br>- FRS<br><br>- MMPI 2<br>- EDI 2, Body dissatisfaction scale<br>- WELSQ<br>- BIAQ	VR tool associated with CBT sessions (ECT) and case study	with CBT + weekly individual psychotherapeutic sessions + psychotherapeutic sessions in biweekly group<br><br>- Immersive Material (HDM), VEBIM-2<br><br>- Pre / post treatment assessment	- Reduction of the dissatisfaction of the body image (BSS, CDRS, FRS, EDI-2)<br>- Reduction of avoidance behaviors, neglect of body care (BIAQ)<br>- High motivation for change

Participants: AN: Anorexia nervosa; BN: Bulimia Nervosa; ED: Eating Disorders; EDNOS: Eating Disorder Not Otherwise Specified

Evaluation Scales: AI: Assertion Inventory; ASI: Appearance Schemas Inventory; BAI: Beck Anxiety Inventory; BASS : Body Areas Satisfaction Scale; BAT : Body Attitude Test; BCRS : Breast/ Chest Rating Scale; BDI: Beck Depression Inventory; BES: Body Esteem Scale; BIAS : Body Image Assessment Software; BIAQ : Body Image Avoidance Questionnaire; BIATQ : Body Image Automatic Thoughts Questionnaire; BIS-11: Barratt Impulsiveness Scale-version 11; BITE : Bulimic Investigatory Test Edinbury; BMI: Body Mass Index; BSQ: Body Shape Questionnaire; BSS : Body Satisfaction Scale; BULIT-R: Bulimia test-revised; CDB : Barcelona Depression Questionnaire; CDRS : Contour Drawing Rating Scale; CPT-II: Conner's Continuous Performance Test II; DASS: Depression, Anxiety and Stress Scale; DEBQ: Dutch Eating Behavior Questionnaire; DIET: Dieter's Inventoy of Eating Temptations; EAT-26 : Eating Attitude Test 26; EDEI : the eating Disorder Examination Interview 16 OD; EDI: Eating Disorder Inventory ; EDE-Q: Eating Disorders Examination Questionnaire; EOQ: Emotional Overeating Questionnaire; EPI: Eysenck Personality Inventory; FCQ-T/S: State and Trait Food Craving Questionnaires; FRS : Figure Rating Scale; IGT: Iowa Gambling Task; ITC-SOPI: The revised version of the ITC-Sense of Presence Inventory; MMPI 2: Minnesota Multiphasic Personality Inventory 2; PANAS: the Positive and Negative Affect Schedule; PBEBI: Precipitating binge eating behavior inventory; RAS: Rathus Assertion Schedule; RJPQ: Reality Judgment and Presence Questionnaire; RS: Restraint Scale; RSEQ: Rosenberg Self-Esteem Questionnaire; SCL-90-R: Symptom Check List-90 items-Revised; SIBID : Situational Inventory of Body Image Dysphoria; STAI: State and Trait Anxiety Inventory; TCI-R: Temperament and Character Inventory-Revised; TFEQ: Three Factors Eating Questionnaire; URICA: University of Rhode Island Change Assessment Scale; WELSQ: Weight Efficacy Life-Style Questionnaire.

Protocol: CBT: Cognitive-Behavioral Therapy; ECT: VR-Enhanced Cognitive behavior Therapy; HDM: Head mounted Display; NS: Not Specified; RCT: Randomized controlled trials; VEBIM: Vitual Environnement for Body Image Modification; VR: Virtual Reality; VREDIM: Virtual Reality for Eating Disorders Modification
